# Stress distribution in fixed mandibular prostheses fabricated by CAD/CAM and conventional techniques: Photoelastic and strain gauge analyses

**DOI:** 10.4317/jced.56067

**Published:** 2019-09-01

**Authors:** Rodrigo-Antonio de Medeiros, Daniela-Micheline dos Santos, Aldiéris-Alves Pesqueira, Marcio Campaner, Sandro-Basso Bitencourt, Emily-Vivianne-Freitas da Silva, Marcelo-Coelho Goiato

**Affiliations:** 1 DDS, MSc, PhD. UNIEURO University Center, Brasília, Federal District, Brazil; 2 DDS, MSc, PhD. Department of Dental Materials and Prosthodontics, Sao Paulo State University (UNESP), Araçatuba, Sao Paulo, Brazil; 3 DDS, MSc. Department of Dental Materials and Prosthodontics, Sao Paulo State University (UNESP), Araçatuba, Sao Paulo, Brazil

## Abstract

**Background:**

The aim of this study was to evaluate the distribution of stress in complete fixed mandibular prostheses with infrastructures (IE) fabricated with different materials and techniques, under compressive force.

**Material and Methods:**

A model of an edentulous mandible, which received five 4x11 mm external hexagon implants between the mental foramens, was fabricated. The groups were divided into: Group I - IE in nickel-chromium with an acrylic resin occlusal coating; Group II – IE in nickel-chromium with a ceramic occlusal coating; Group III – IE milled in zirconia with a ceramic coating. For the photoelastic methodology, 70 N axial loads were applied in three regions. Photographic images were taken and analyzed according to the number of high-intensity fringes. For the strain gauge methodology, the measurement of stresses was performed in two distinct regions. The same compression tests described earlier were then performed. The registered stress values were grouped in tables and submitted to two-factor variance analysis (ANOVA) and the Tukey test with 5% significance.

**Results:**

The results of the two methodologies demonstrated smaller stress values for Group I, when compared to the other groups.

**Conclusions:**

It was possible to conclude that the complete fixed prostheses, with infrastructures cast in metal and acrylic occlusal coating, demonstrated better biomechanical results.

** Key words:**Dental implants, mandibular prosthesis implantation, biomechanics.

## Introduction

Before the use of osseointegrated implants, the only available treatment option for fully edentulous patients was the complete muco-supported prosthesis.1 However, in some cases, even when executed well, the prosthesis does not totally restore the functional capacity of patients, due to the qualitative and quantitative reduction of masticatory efficiency (1).

In addition, it is known that the muco-supported mandibular prostheses present a high rate of patient dissatisfaction, due principally to instability and lack of retention, leading to a decrease of self-confidence, quality of life, and social contact (2). Thus, the prosthetic rehabilitation of these flanges with dental implants improves the oral function and benefits bone maintenance (3).

For edentulous patients, a Branemärk-type complete fixed mandibular prosthesis is a predictable and favorable treatment which restores functional capacity and presents a high rate of clinical success (4). In this context, it could be considered the best form of rehabilitation for fully edentulous mandibles, based on patient satisfaction evaluations (5).

The conventional complete fixed prosthesis consists of a bar cast in implant-supported metal alloy with an acrylic resin or porcelain coating. However, the frequent need to section and solder, to obtain a passive adaptation, is one of the disadvantages of this technique (6). To solve this problem, the great evolution in dental treatment is the use of the CAD/CAM (computer-aided design and computer-aided manufacturing) (7) principle of engineering, allowing milling of complete fixed prosthesis infrastructures in titanium, ceramic, and cobalt-chromium, aiming to provide the greatest adaptation possible (8).

Various published studies have the objective of evaluating the adaptation of these milled prostheses, with the goal of minimizing the bacterial microinfiltration, and consequently the risk of peri-implantitis (9). However, there are no known published studies that evaluated the load distributions of these complete fixed mandibular prostheses with the milled structure, which is of fundamental importance for correct planning and long-term success. The optimization of masticatory load distribution by means of prostheses, and those for implants and bone support, must be performed, and respect the physiologic limits so that the tissue response will not be adverse (10). In an edentulous mandible, the bone base is less dense, and thus, the bone is no longer capable of accepting physiologic forces, in addition to not possessing the periodontal ligament; the principal mechanism of absorption of these forces (11).

Therefore, the aim of this study was to evaluate the distribution of stress, by means of the photoelastic methodology and strain gauge, in complete fixed mandibular prostheses with infrastructures fabricated with different materials and techniques, submitted to the force of compression. The null hypothesis of this study is that there will be no difference in the distribution of stress between the conventional complete fixed prostheses and those milled in zirconia, and between the coating materials.

## Material and Methods

An experimental cast of type IV plaster (Durone; Dentsply Ind Com Ltda) of an edentulous mandible received 5 external hexagon (EH) analogs (TitamaxTi; Neodent) distributed between the mental foramen, with a 10-mm distance between the center of the most anterior analog and the line that passes through the distal of the 2 most distal analogs (distance A-P). The analogs were positioned at the level of the resin corresponding to the level of the bone ridge, and equidistant between themselves, with the aid of a delineator to obtain the parallelism between the implants.

Transfer squares were screwed to the analogs and unitied with dental floss and acrylic resin (Duralay, Reliance Dental Co). This cast was molded with silicone fluid (Silicone fluid; Sapeca Artesanato) for later obtainment of the photoelastic cast (12). In the fabricated cast, five 4x11 mm external hexagon implants were screwed to the transfer squares.

After the adaptation of the implants to the transfers, PL-2 photoelastic resin (Vishay Measurements Group Inc.) was manipulated according to the manufacturer’s recommendations, poured over the mold, and placed in a closed recipient under a pressure of 40 lbf/pol2 for 24 hours for the removal of internal bubbles. After the polymerization of the PL-2 resin, the cast was separated carefully from the mold and submitted to finishing and polishing with fine granulation sandpaper (600, 800, 1200, and 1500) (CarbiMet 2; Buehler)(13).

The groups were divided according to  Table 1. Twenty-one prostheses were fabricated and divided between the groups (n=7). The groups being: metallic cast infrastructures in nickel-chromium (Fit Cast-SB; Talmax) with artificial teeth in acrylic resin (Trilux Ruthinium; VIPI Produtos Odontológicos) (GI); metallic cast infrastructure in nickel-chromium (Fit Cast-SB; Talmax) with ceramic coating (Vita VM13; Vita) (GII); infrastructures milled with yttrium-oxide stabilized zirconia (Zirkonzahn) with ceramic coating (Vita VM13; Vita) (GIII). The infrastructures of all prostheses were the same distance in relation to the photoelastic cast ridge, and the crowns were fabricated with the same dimensions. The length of the cantilever in all prostheses was 15 mm.

**Table 1:**
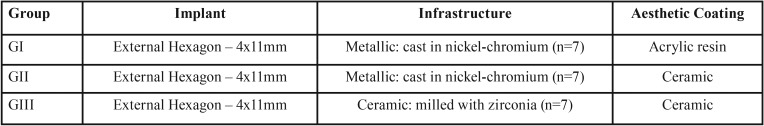
Division of tested groups.

-Photoelastic methodology

For the photoelastic methodology, the photoelastic model with the prostheses was inserted individually in a circular polariscope adapted to a universal testing machine (EMIC DL 300; EMIC). Axial loads of 70N were applied to fixed points and standardized in the most anterior region of the implant, between incisors, and in the center of the first molar of each side of the prosthesis.

The resulting stress in all of the areas of the photoelastic model were monitored, photographically registered, and subsequently, visualized in a computer by a graphics program (Adobe Photoshop CS6, Adobe Systems), with the intent of facilitating visualization, comprehension, and interpretation, both in the localization and intensity of the distributed stresses around the implants and the bone tissue. The photographic registers were qualitatively analyzed by an evaluator, according to the number of high intensity fringes (green-pink transition) (12). Zaparolli *et al.* (14) attributed values to transition fringes according to  Table 2. The evaluator was shielded, not knowing which group each photographic register belonged to, avoiding bias in the results. A summary of the number of high intensity fringes for each group was performed.

**Table 2:**
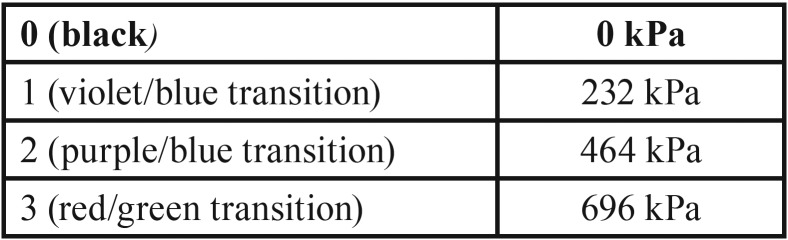
Fringe order and the corresponding stress value (20).

-Strain gauge methodology

The analysis by strain gauge methodology was performed later. The measurement of the stresses was performed in 2 distinct regions using the photoelastic model fabricated previously. For the first region, two electric strain gauges (PA06060BA; Excel sensors Ind. Com. Exp. Ltda) were positioned horizontally in the mesial and distal region of the implants, directly over the marginal ridge of the photoelastic model.

For the second region, the photoelastic resin was removed from the vestibular and lingual surface of each implant, using a handheld spherical dental bur and Maxicut (Edenta 1503; Edenta), with 1 mm of resin maintained intact, where the strain gauges were vertically fixed, after the finishing and polishing. Since it is not possible to assess the stress directly over the implant, it was assumed that the stress generated on the resin around the implants would represent the stress induced to the bone (15).

Each strain gauge was mounted in a one-quarter Wheatstone bridge configuration and had its signals digitalized by a data acquisition system (ASD 2002; Lynx Tecnologia Eletrônica Ltda). The same axial cargo tests used in the photoelastic methodology were used, and each load was applied 5 times on each standardized point for each prosthesis (most anterior implant region and first molar of each side). The mean of these 15 applications denominated the value for each prosthesis.

-Statistical Analyses

The registered stress values (microstrains) were grouped in tables and submitted to statistical analysis. Statistical analysis of results was performed using SPSS 11.5.0 software (SPSS, Chicago, Illinois 60606, USA). The Shapiro-Wilk test was applied to verify normal distribution of numerical numbers. The two-way variance analysis (ANOVA) and post-hoc Tukey test were used for the analysis, with a 5% significance level. The methodology was reviewed by a statistician.

## Results

**Figure 1:**
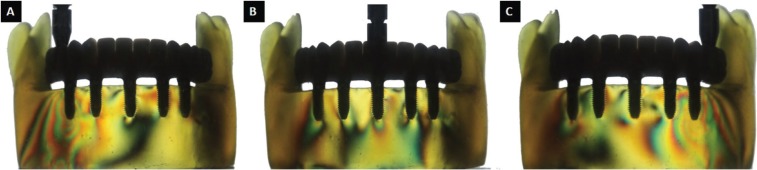
Axial load application in prostheses with metallic infrastructure and acrylic coating (GI). A) Right molar. B) Most anterior implant region, between incisors. C) Left molar.

The results of the 2 methodologies were similar. Through the photoelastic methodology, it was observed that the prostheses with a metallic infrastructure and an aesthetic acrylic resin coating (GI) presented the smaller number of high intensity fringes, as demonstrated in  Table 3 and in Figures 1 and 3. All fringes were encountered in the apexes of the implants.

**Table 3:**
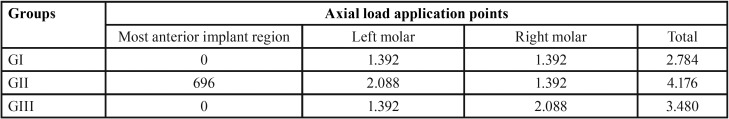
Results of high intensity fringe number count (green-pink transition = 696 kPa) of photoelastic methodology.

**Figure 2:**
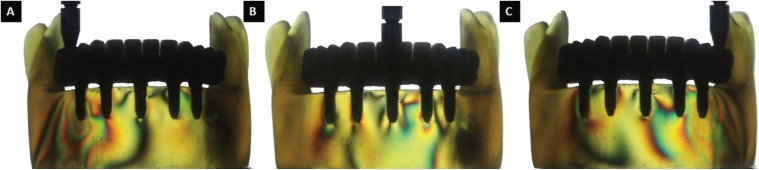
Axial load application in prostheses with metallic infrastructure and ceramic coating (GII). A) Right molar. B) Most anterior implant region, between incisors. C) Left molar.

**Figure 3:**
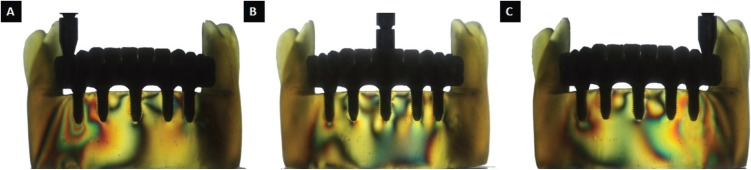
Axial load application in prostheses with infrastructure milled in zirconia and ceramic coating (GIII). A) Right molar. B) Most anterior implant region, between incisors. C) Left molar.

Through the strain gauge methodology, the interaction between the prosthesis factors and strain gauge region significantly interfered in the mean values of stress measured by two-way ANOVA ( Table 4).

**Table 4:**
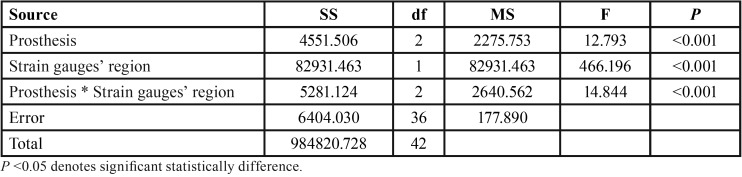
Two-way ANOVA of stress values measured in models studied in strain gauge analysis.

There was a significant statistical difference (*P*<0.001) between the 2 regions where the strain gauges were inserted, in which greater stress values were encountered in the mesial/distal region in all of the prosthesis types when compared to the vestibular/lingual region. The mean stress values of each type of prosthesis and the strain gauge regions are found in  Table 5.

**Table 5:**
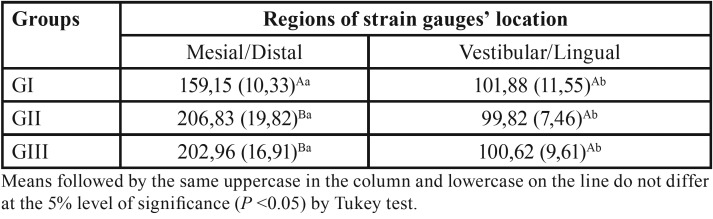
Mean values and standard deviation of stress values (in microstrains) for each group, according to regions of strain gauge location.

In relation to the types of prosthesis, there was a significant statistical difference between the groups only when the strain gauges were inserted in mesial and distal regions of the implants. In this analysis, G1 presented smaller stress values when compared to GII and GII (*P*<0.001). There was no significant statistical difference between GII and GIII (*P*=0.591). The results were reviewed by a statistician.

## Discussion

The null hypothesis was rejected since the prostheses with a metallic infrastructure and aesthetic acrylic resin coating presented smaller numbers of high intensity fringes and stress (microstrains), first through the photoelastic methodology, and then the strain gauge methodology.

The present study evaluated the dissipation of loads for the bone/implant interface by means of 2 methodologies: photoelastic and strain gauge analysis. Despite being different methodologies, both present similar results, demonstrating that Branemärk-type complete fixed mandibular prostheses exhibit greater values of stress when the ceramic occlusal coating was used, and that the material used for the fabrication of the infrastructure, whether cast in nickel-chromium or milled zirconia, did not present a difference in the load dissipations.

Prostheses with rigid infrastructures, such as nickel-chromium (elastic modulus: 200 GPa) and zirconia (elastic modulus: 205 GPa), transmit less stress to the implant and prosthetic components, when compared to less rigid infrastructures, such as titanium (16). Nonetheless, this variation of infrastructure material rigidity does not demonstrate a significant effect on the stress values in the marginal bone around the implants (17). In the present study, rigid infrastructures were used, demonstrating that the occlusal coating had a greater influence on the load dissipations than the infrastructure.

Smaller values of stress were encountered for the complete fixed mandibular prostheses with the metallic infrastructure and aesthetic coating of artificial acrylic teeth. Considering the occlusal coating, this result corroborates with Meriç *et al.* (18) and Ciftci *et al.* (19), in studies of finite elements, where screwed prostheses of three elements with acrylic resin presented 25% less stress than those of porcelain. This material possesses less elastic modulus (2.26 GPa) than ceramic (70 GPa), causing the absorption of the forces and the transference of stress to the support bone (19).

In controversy, Santiago-Junior *et al.* (20) concluded there was no difference in the stress distribution around the bone/implant interface when different coating materials were analyzed, such as acrylic resin and porcelain, in screwed single-unit prostheses. For these authors, larger diameter implants presented smaller stress value, and that is considered more important than the occlusal coating material. One of the motives for the results being different could have been the study methodology, which used pre-established numeric data in the computational program, and the use of virtual models, different from the present study, as well as the type of prosthesis studied.

Ferreira *et al.* (17) did not find a difference between the infrastructures tested (titanium, gold, chrome-cobalt, nickel, chromium, and silver-palladium) and the occlusal coating in the dissipation of loads for the marginal bone. However, in relation to the occlusal coating material, complete fixed mandibular prostheses fabricated with porcelain teeth, independent of the infrastructure material, presented 50% less transmitted stress to the infrastructure when compared to acrylic resin teeth. According to the authors, acrylic resin possesses a low elastic modulus which could result in a greater deflection, resulting in greater stress values for the infrastructure. Porcelain, which possesses a greater elasticity modulus and is more rigid, also possesses elevated resistance to bending, allowing the dissipation of loads to this prosthetic structure.

Thus, it is possible to observe that the influence of the coating material is still a subject that must be studied considerably, through the different methodologies, for the acquisition of data that provides better clinical results. Additionally, it is also necessary to take into consideration the results of the clinical studies on these materials used for the fabrication of infrastructures and occlusal coating.

Therefore, despite the present study having encountered smaller values of stress for the bone/implant interface, acrylic resin possesses mechanical property deficiencies, such as low resistance to abrasion and fracture, which could result, during mastication, in the exposure of the infrastructure or masticatory deficiency due to the loss of vertical dimension over time (12). Ventura *et al.* (21) founded that 40% of the prostheses presented failures (fracture of artificial teeth) which were related to the type of gender, opposite arch, size of the cantilever, and mechanical retention of the metallic infrastructure. Male patients with natural teeth in the opposite arch, long cantilevers, and metallic infrastructure without mechanical retention presented greater fracture rates of the acrylic resin (21).

When using the ceramic occlusal coating, the most encountered problem is the fracturing of this material, when placed in occlusion, since the functional load added to the residual stress of the fabrication process could cause the chipping of the porcelain (22). In addition, being a very rigid material, ceramic could cause wear of the antagonist tooth, making it necessary to always verify the occlusion and occlusal adjustments.

Despite the results with greater stress values having been encountered for the prostheses with infrastructure milled in zirconia and aesthetic porcelain coating, the published literature reports that infrastructures milled by CAD/CAM technology present better marginal adaptation to the implant (23), preventing the accumulation of bacterial biofilm in the prosthesis/implant interface, decreasing the peri-implantar inflammation risk, and consequently, minimizing the marginal bone loss (24). Therefore, randomized clinical studies should be realized to confirm if this significant statistical difference between the tested groups results in greater bone loss for patients rehabilitated with the metal-ceramic complete fixed prostheses, or with infrastructure milled in zirconia with aesthetic ceramic coating.

Additionally, the published studies that evaluated the properties of materials used for fabrication of the prostheses are in vitro, making caution necessary in the interpretation of the results, since a significant statistic difference in these studies might not demonstrate a considerable difference in the clinical success rate of the rehabilitations. Vizcaya *et al.* (25) evaluated complete fixed maxillary and mandibular prostheses with infrastructures milled in zirconia, demonstrating a 100% success rate in the prostheses and without the loss of any implant (25).

The present study also evaluated the load applications in 2 distinct regions, since greater stress values, through the strain gauge methodology, were encountered on the mesial and distal surfaces of the ridge of the studied model, when compared to the vestibular and lingual regions, which in the study simulate the load transmitted to the adjacent bone of the dental implant. These results demonstrate that biomechanical principles must be respected, such as small cantilevers, a greater number of implants, and an increase in the implant diameters, to avoid greater loads on the marginal bone ridge. The stresses in this region are more damaging for marginal bone loss than stresses found in the implant body and its apical region.

Despite being an in vitro study, the results of the present study contribute to the acquisition of knowledge about how to avoid an increase in the stresses of the implant/bone interface in clinical conditions. It is always necessary to analyze the factors that could lead to marginal bone loss, and consequently could lead to clinical failure, with one of the factors being stress transmitted to the system. Enhancing the load dissipation and avoiding marginal bone loss permits rehabilitation longevity, bringing greater satisfaction and quality of life to the patient.

The present study is an in vitro work presenting limitations as the low fidelity of the photoelastic resin simulating the variations in the bone tissue, limiting the results in the results. More biomechanical evaluation methods might help clarify the results, and, randomized clinical trials should be performed to evaluate whether the biomechanical difference between the groups evaluated leads to marginal bone loss of dental implants. It is of fundamental importance to transmit its results to *in vivo* studies with the objective of demonstrating if the difference present in the *in vitro* study also occurs *in vivo*, in relation to clinical longevity.

## Conclusions

Thus, based on the results obtained, it is possible to conclude that complete fixed prostheses with cast metal infrastructure and acrylic occlusal coating demonstrate better biomechanical results. When using ceramic coating material, there is no difference in distribution of stress, independent of the material or technique of infrastructure fabrication. The present in vitro study has limitations; therefore, more in vitro and in vivo studies should be performed with the aim of greater longevity of oral rehabilitations.
